# Predictive value of urinary cell cycle arrest biomarkers for all cause-acute kidney injury: a meta-analysis

**DOI:** 10.1038/s41598-023-33233-9

**Published:** 2023-04-13

**Authors:** Feng Huang, Yan Zeng, Linghai Lv, Yaoyao Chen, Yan Yan, Laimin Luo, Rong Pan, Jiaming Jiang, Xin Wei

**Affiliations:** 1 Department of Nephrology, The First Affiliated Hospital of Nanchang University, Nanchang, 330006 Jiangxi China; 2 Nanchang University, Nanchang, 330006 China; 3 Shangrao Guangxin District People’s Hospital, Shangrao, 334100 Jiangxi China

**Keywords:** Diagnostic markers, Acute kidney injury

## Abstract

The cell cycle arrest markers tissue inhibitor metalloproteinases-2 (TIMP-2) and insulin-like growth factor-binding protein 7 (IGFBP7) have been identified as potential biomarkers of acute kidney injury (AKI) in critically ill adults in intensive care units and cardiac surgery-associated AKI (CSA-AKI). However, the clinical impact on all-cause AKI remains unclear. Here, we report a meta-analysis performed to evaluate the predictive value of this biomarker for all-cause AKI. The PubMed, Cochrane, and EMBASE databases were systematically searched up to April 1, 2022. We used the Quality Assessment Tool for Diagnosis Accuracy Studies (QUADAS-2) to assess the quality. We extracted useful information from these studies and calculated the sensitivity, specificity, and area under the receiver operating characteristic curve (AUROC). Twenty studies with 3625 patients were included in the meta-analysis. The estimated sensitivity of urinary [TIMP-2] × [IGFBP7] in the diagnosis of all-cause AKI was 0.79 (95% CI 0.72, 0.84), and the specificity was 0.70 (95% CI 0.62, 0.76). The value of urine [TIMP-2] × [IGFBP7] in the early diagnosis of AKI was assessed using a random effects model. The pooled positive likelihood ratio (PLR), negative likelihood ratio (NLR), and diagnostic odds ratio (DOR) were 2.6 (95% CI 2.1, 3.3), 0.31 (95% CI 0.23, 0.40), and 8 (95% CI 6, 13), respectively. The AUROC was 0.81 (95% CI 0.78–0.84). No significant publication bias was observed in eligible studies. Subgroup analysis indicated that the diagnostic value was related to the severity of AKI, time measurement, and clinical setting. This study shows that urinary [TIMP-2] × [IGFBP7] is a reliable effective predictive test for all cause-AKI. However, whether and how urinary [TIMP-2] × [IGFBP7] can be used in clinical diagnosis still requires further research and clinical trials.

## Introduction

Acute kidney injury (AKI) is a clinical syndrome with a high incidence of extensive renal damage, which can cause abnormalities in renal structure and function. With improvements in the understanding and diagnosis of AKI and the aging of the population, the clinical incidence of AKI has increased significantly in recent years. A systematic review that included 49 million patients identified that AKI occurred in one in five adults and one in three children hospitalized with acute illness^1^. Furthermore, multiple studies have found that AKI is an important risk factor for higher morbidity and mortality. The overall mortality rate of AKI is close to 25%, and the death rate of severe cases is over 50%. In addition, patients with AKI are at high risk of developing chronic kidney disease (CKD), which places a huge burden on medical and health care^2,3^. Therefore, it is crucial to address the early and potentially reversible stages of acute kidney injury. Typically, AKI is diagnosed by creatinine-based guidelines that rely solely on guidelines for increased serum creatinine or decreased urine volume^4,5^. However, serum creatinine concentration does not increase until approximately half of the kidney function is lost, and it may be affected by sex, muscle mass, and the patient’s hydration status^6,7^. Although elevated blood urea nitrogen and serum creatinine levels are the most widely accepted biomarkers of AKI, their increase may be delayed by 24–72 h following AKI induction.

New biomarkers will help in the early diagnosis of AKI, making the identification of these biomarkers of major clinical interest. Recently, the new biomarkers tissue inhibitor metalloproteinases-2 (TIMP-2) and insulin-like growth factor-binding protein 7 (IGFBP7) have been proposed for the early detection of cardiac surgery-associated acute kidney injury (CSA-AKI)^8^. Both are involved in the G1 cell cycle, which is a known mechanism of AKI^9^. In human kidneys, TIMP-2 is expressed in the distal nephron, whereas IGFBP7 is mainly expressed in the proximal tubule, and both markers can be detected in urine samples^10^. In 2014, the Nephrocheck™ test, which calculates [TIMP-2] × [IGFBP7] concentrations, was developed and approved by the Food and Drug Administration (FDA) for use in intensive care unit (ICU) patients to predict the risk of developing moderate to severe AKI^11^. Since then, an increasing number of studies have evaluated the clinical application of [TIMP-2] × [IGFBP7] in AKI of different etiologies. Thus, we performed a meta-analysis to determine the clinical value of urinary [TIMP-2] × [IGFBP7] levels in all cause-AKI.

## Methods

### Search strategy

A complete meta-analysis protocol was constructed in adherence with the Preferred Reporting Items for Systematic Reviews and Meta-Analyses (PRISMA) standards. We performed a comprehensive literature search of the MEDLINE, PubMed, and EMBASE databases from 2013 to April 2022 to identify relevant articles. The literature search included the keywords and MeSH (medical subject headings) terms “acute kidney injury”, “acute renal failure”, “acute tubular necrosis”, “TIMP-2”, or “Tissue Inhibitor of Metalloproteinase-2”, “IGFBP7”, “IGF-binding protein 7”, “insulin-like growth factor binding protein 7”, with no language restrictions. The full search strategy is provided in the Supplementary Document. The search strategy was manually adapted according to the citation lists of the retrieved articles for the sensitivity analysis. The reference lists of selected studies were manually searched to identify potentially relevant citations.

### Study selection

Two investigators independently evaluated all identified articles for eligibility and inclusion. Any disagreements were resolved by consultation with a third investigator. Although there were no initial language restrictions, for the full-text review and final analysis, we included only articles published in English. Studies included met the following criteria: (1) original study; (2) urinary [TIMP-2] × [IGFBP7] as biomarkers for the early diagnosis of AKI; and (3) studies with mandatory data from which true-positive (TP), false-positive (FP), false-negative (FN), and true-negative (TN) could be found or calculated. The exclusion criteria were as follows: (1) studies from conference abstracts, guidelines, letters, editorials, or reviews; (2) studies without sufficient data for analysis, even after contacting the authors; and (3) studies with duplicate data reported in other studies.

### Data extraction and quality assessment

Data on the patients and study characteristics were collected and entered into a database to assess study eligibility. If eligible, a standardized data extraction sheet was used for data extraction. The following data were extracted: first author, year of publication, publication data, study design, population type, age, sample size, test method, timing of measurement, AKI definition, sample storage, TP, FP, FN, TN, sensitivity, specificity, and area under the receiver operating characteristic curve (AUROC). We assessed the methodological quality using the Quality Assessment Tool for Diagnosis Accuracy Studies (QUADAS-2)^12^. Any discrepancies that arose from the study selection, data extraction, and quality assessment were resolved by discussion to reach a final consensus.

### Statistical analysis

All statistical analyses were conducted using Review Manager 5.4 (RevMan; The Cochrane Collaboration, Oxford, UK) and STATA 14.0 software (Stata Corp, LP, College Station, TX, USA). A random-effects model or fixed-effects model was constructed to estimate the pooled sensitivity, specificity, pooled positive likelihood ratio (PLR), negative likelihood ratio (NLR), and diagnostic odds ratio (DOR) with 95% CI. The model selection was based on the heterogeneity of the included studies^13^. Diagnostic accuracy analysis was consistent with the summary receiver operating characteristic (SROC) curve and area under the curve (AUC) of the SROC. The heterogeneity induced by the threshold effect was set at P < 0.05. Heterogeneity in the meta-analysis represented the degree of variation in the study results, and was assessed using Cochran’s Q test and I^2^ test^14^. Cochran’s Q test indicated heterogeneity at P < 0.10, and I^2^ > 50% was considered an indication of significant heterogeneity. A useful predictor of AKI risk was defined as an AUROC of > 0.7 and P < 0.05. The Fagan nomogram was used to calculate post-test probability (PTP). Deeks’ funnel plot asymmetry test was used to check for publication bias using STATA 14.0^15^. All statistical tests were 2-sided, and statistical significance was set at P < 0.05.

### Ethics approval and consent to participate

The local Institutional Review Board deemed the study exempt from review.

## Results

### Search results

**Figure 1:**
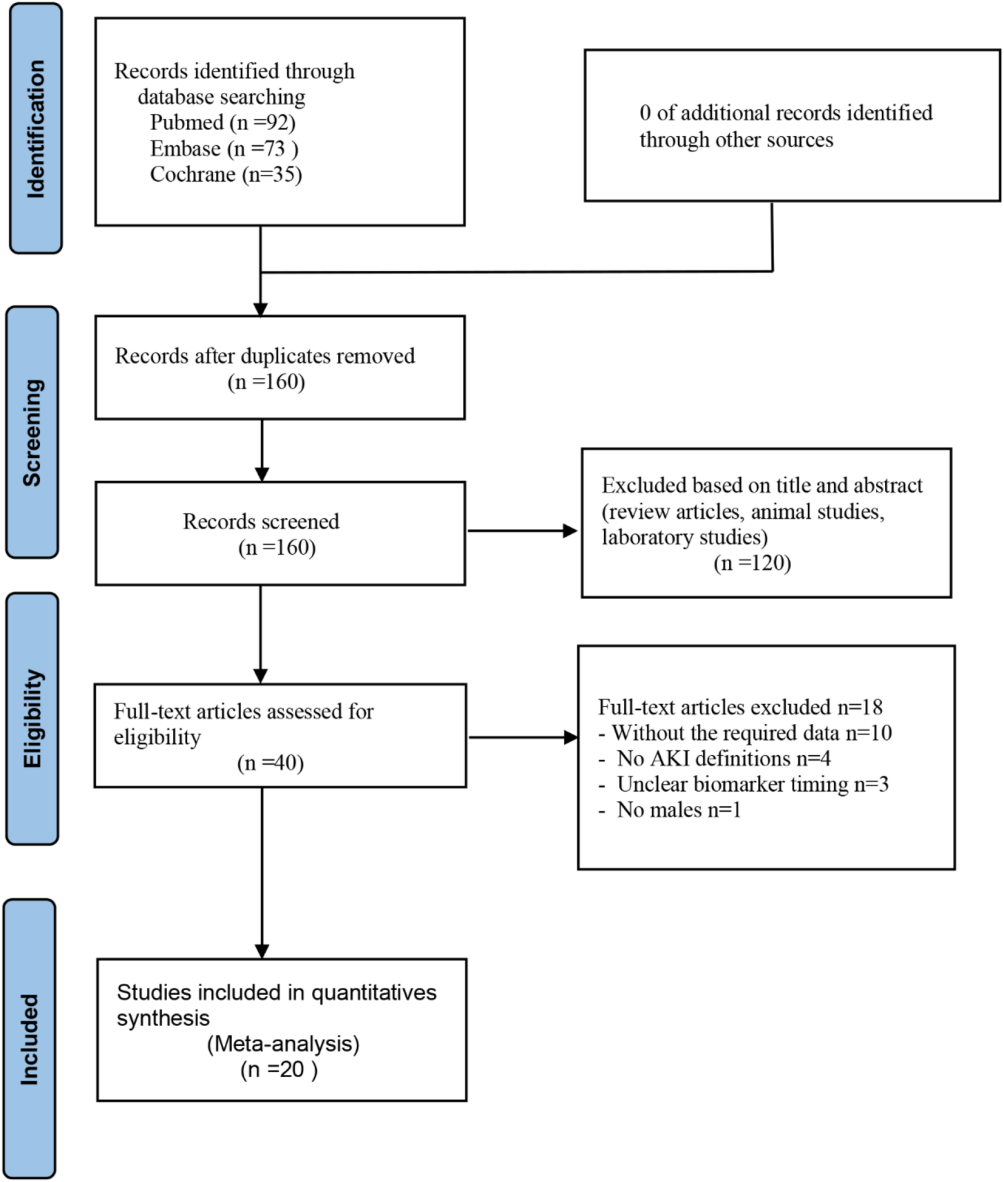
Flow chart of study selection.

A total of 200 articles were preliminarily identified through the search. First, 40 studies were removed after duplicates were identified. We excluded 120 studies by screening the titles and abstracts. After reviewing the full text of the remaining studies, a further 18 publications were excluded: 10 did not have the required data, 4 had no definition of AKI, 3 had unclear measurement time of biomarkers, and 1 had no male subjects. Moreover, two studies were unable to extract a 2 × 2 table data, but only AUROC prediction values^16,17^. Finally, 20 studies were included in the quantitative analysis (Fig. 1)^10,11,18–35^.

### Study and patient characteristics

All studies were published between 2013 and 2022, and a total of 3625 patients were included in this meta-analysis. Table 1 presents the characteristics of the included studies. One of the included studies was conducted on infants^30^, and the remaining 19 studies were conducted on adults. The included studies were conducted on patients with different AKI settings. Ten studies focused on patients who had undergone cardiac surgery, 5 included critically ill patients, 2 included emergency department (ED) patients, 2 included trauma patients, and 1 included patients with out-of-hospital cardiac arrest. It is worth noting that these articles had different definitions of AKI. Urinary [TIMP-2] × [IGFBP7] levels were measured by a commercial enzyme-linked immunosorbent assay (ELISA) in two studies, and the remaining 18 studies were measured using the FDA-approved NephroCheck Test.

**Table 1:** Characteristics of Incloud studies.

Study	Country	Study design	Population type	Age (y)^a^	Males	Sample^a^	Clinical department	AKI definition	Detection method	Sample storage (°C)
Kashani (2013)^10^	USA	PC	Adullts	65/64	449	101/627	ICU	RIFLE^d^	NephroCheck	− 70 °C
Bihorac (2014)^18^	USA	PC	Adullts	62/63	219	71/337	ICU	RIFLE^d^	NephroCheck	− 70 °C
Meersch (2014)^20^	Germany	PC	Adullts	70/72	36	26/24	CPB	RIFLE^d^	NephroCheck	− 70 °C
Hoste (2014)^19^	Belgium	PC	Adullts	64/65	87	27/126	ICU	KDIGO 2–3	NephroCheck	− 70 °C
Pilarczy (2015)^21^	Germany	PC	Adullts	76.2/68.8	48	6/54	CABG	KDIGO 2–3	NephroCheck	NR
Dusse (2016)^22^	Germany	PC	Adullts	81.4/80.7	16	8/32	CS	KDIGO 2–3	NephroCheck	NR
Honore (2016)^23^	Belgium	NR	Adullts	64/62	119	40/192	ICU	KDIGO 2–3	NephroCheck	NR
Kimme (2016)^24^	Germany	NR	Adullts	65/63	216	46/252	ED	KDIGO 2–3	NephroCheck	− 70 °C
Finge (2017)^25^	France	PC	Adullts	75/66	69	34/59	CPB	KDIGO^e^	NephroCheck	NR
Oezkur (2017)^11^	Germany	PC	Adullts	NR	NR	35/115	CPB	KDIGO^e^	NephroCheck	NR
Wang (2017)^27^	China	PC	Adullts	64/53	41	20/37	CS	KDIGO^e^	NephroCheck	NR
Mayer (2017)^26^	Switzerland	PC	Adullts	70/66	87	9/101	CPB	KDIGO^e^	NephroCheck	− 70 °C
Zaouter (2018)^29^	France	PC	Adullts	73/71	28	37/13	CS	KDIGO^e^	NephroCheck	NR
Adler (2018)^28^	Germany	PC	Adullts	63/62	44	31/17	OHCA	KDIGO^e^	NephroCheck	NR
Schiefer (2019)^16^	Austria	NR	Adullts	56/58	29	12/28	OLT	KDIGO 2–3	NephroCheck	NR
Fuhrman (2020)^17^	USA	PC	Childrens	114/102mo^b^	6	6/10	LTx	KDIGO^e^	NephroCheck	− 20 °C
Chen (2020)^30^	China	PC	Infants	33.7/35.4wk^c^	117	20/217	NICU	KDIGO^e^	ELISA	− 80 °C
Hatton (2020)^31^	USA	PC	Adullts	40/35	66	39/49	Trauma	KDIGO^e^	NephroCheck	− 80 °C
Sakyi (2021)^32^	Ghana	PC	Adullts	36.8/38.5	66	44/107	Trauma	KDIGO^e^	ELISA	− 80 °C
Waskowski (2021)^33^	Switzerland	PC	Adullts	71.3/68.5	77	31/62	CPB	KDIGO^e^	NephroCheck	NR
Irqsusi (2021)^34^	Germany	PC	Adullts	71.5/64	NR	14/36	CS	KDIGO^e^	NephroCheck	NR
Yang (2022)^35^	Korea	PC	Adullts	65/65	316	59/470	ED	KDIGO^e^	NephroCheck	2–8 °C

*AKI* acute kidney injury, *CABG* coronary artery bypass surgery, *CPB* cardiopulmonary bypass, *CS* cardiac surgery, *ED* emergency department, *ICU* intensive care unit, *LTx* liver transplant, *NICU* neonatal intensive care unit, *NR* not reported, *OHCA* out-of-hospital cardiac arrest, *OLT* orthotopic liver transplantation, *PC* prospective cohort.

^a^A backslash separating 2 values denotes AKI/no AKI; ^b^month; ^c^Gestational age; ^d^RIFLE, risk, injury, failure, loss, end stage kidney disease; ^e^*KDIGO* Kidney Disease: Improving Global Outcomes.

### Definition of AKI

Three studies used RIFLE (risk, injury, failure, loss, end-stage kidney disease)^10,18,20^, and the remaining 17 used the Kidney Disease Improving Global Outcomes (KDIGO) criteria for AKI. Most studies used a minimal threshold for AKI (KDIGO stage 1)^11,25–35^, whereas 5 studies set higher thresholds (KDIGO stage 2–3)^19,21–24^.

### Quality assessment and publication bias

**Figure 2:**
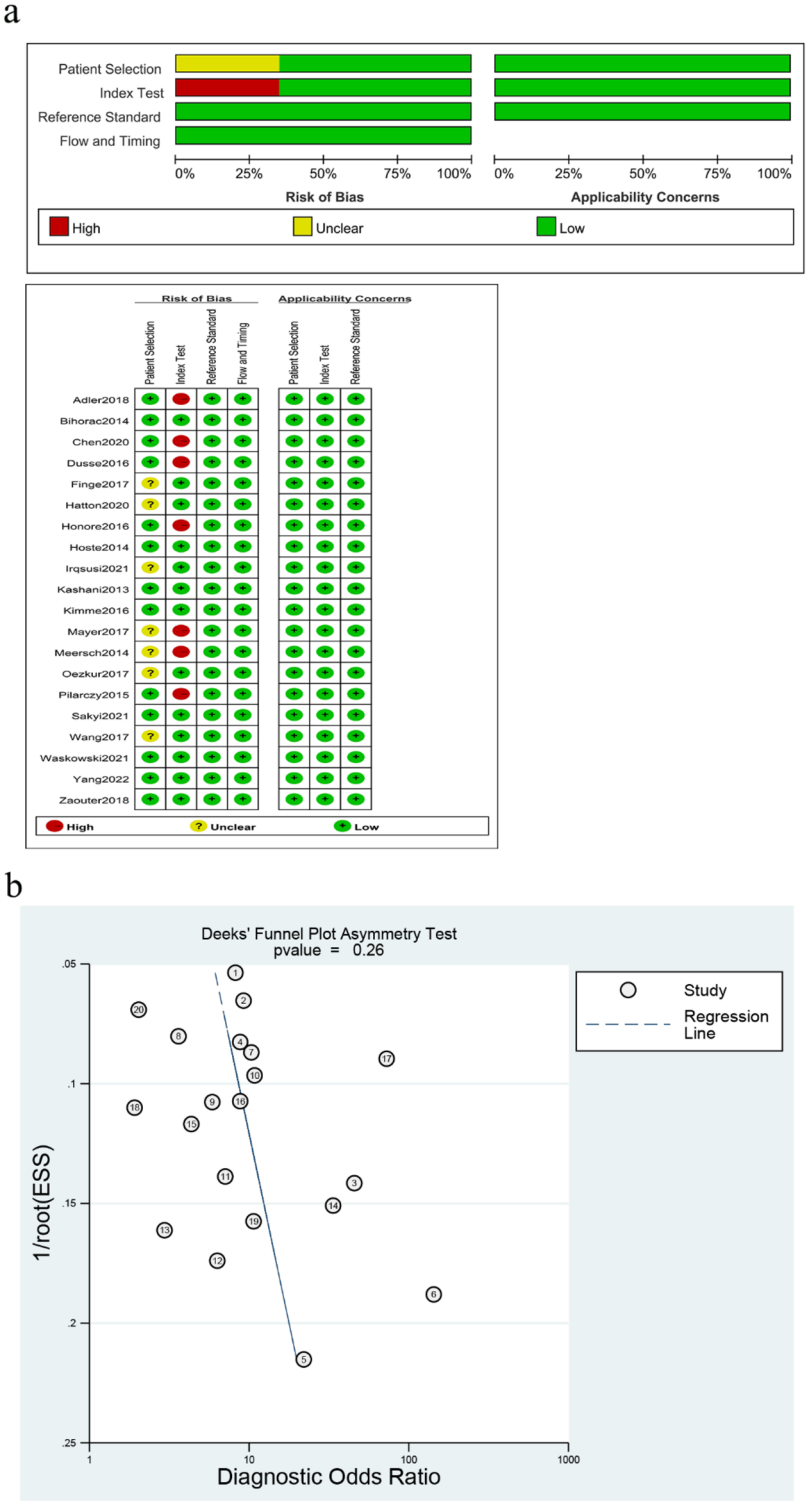
(**a**) Summary of the methodological quality of the studies. ‘−’ in red and ‘+’ in green mean high risk and low risk, respectively. ‘?’ in yellow means unclear risk. (**b**) Deek funnel plot asymmetry test for publication bias.

The quality outcomes of the included studies according to the QUADAS-2 are shown in Fig. 2. Our results revealed that 7 studies did not use a set threshold, resulting in a higher risk in the index test. Concerning the reference criteria, seven studies were assessed as having unclear risk because they did not mention blinding. None of the studies had concerns regarding applicability. Details of the studies are shown in Table 1 and Supplementary Table 1. The Deeks’ funnel plot asymmetry test was used to evaluate publication bias. The finding of P = 0.26 suggests that the likelihood of publication bias is low (Fig. 2).

### Diagnostic value of urinary [TIMP-2] × [IGFBP7] for AKI prediction

**Figure 3:**
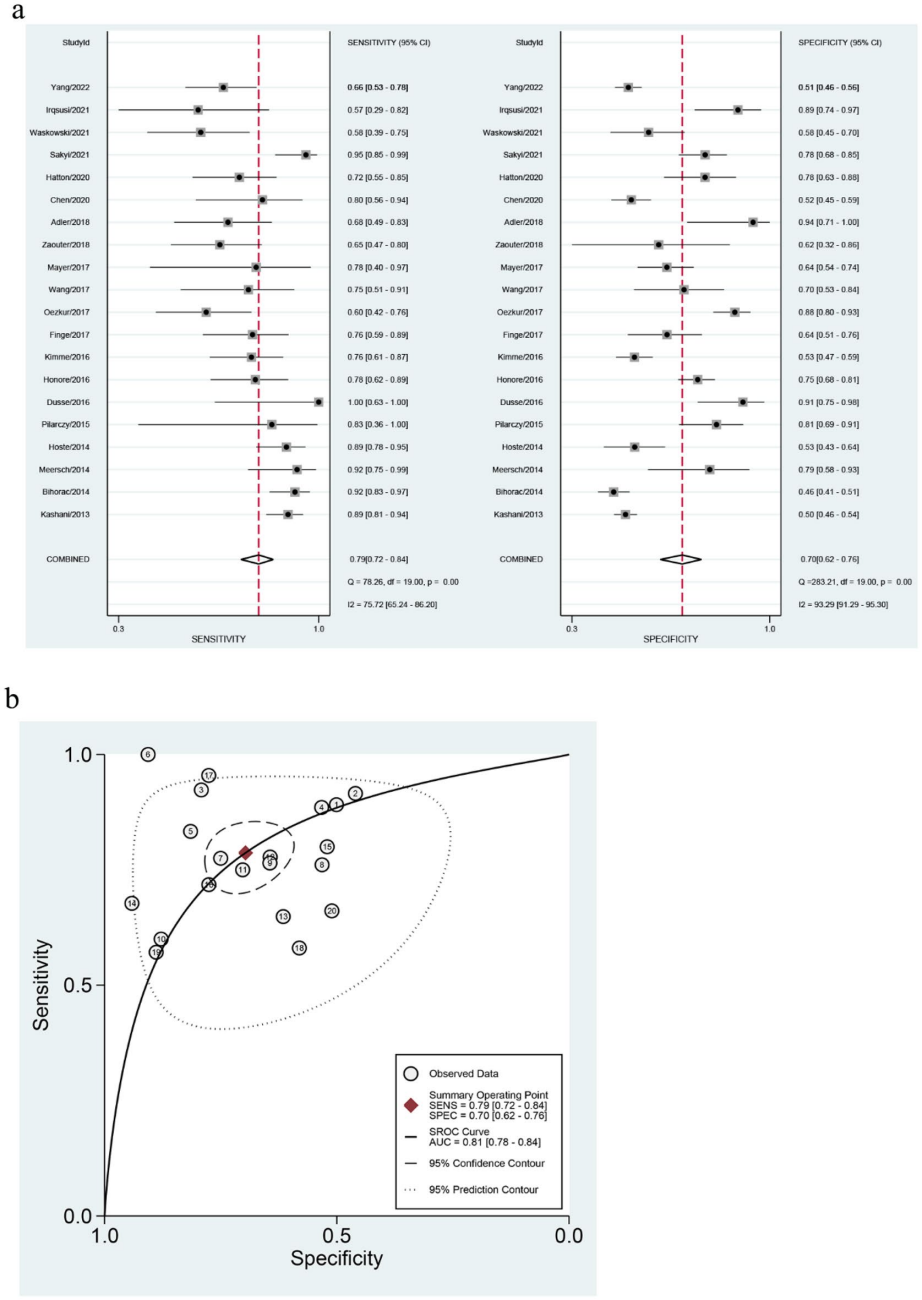
(**a**) Forest plot of the pooled sensitivity and specificity of urine [TIMP-2] × [IGFBP7] in predicting acute kidney injury. (**b**) Summary receiver operating characteristic graph for the included studies. The curve is represented by the straight line; each of the analyzed studies is represented by a circle; the point estimate to which summary sensitivity (SENS) and specificity (SPEC) correspond is represented by the diamond shape, and the respective 95% confidence intervals, by the dashed line, whereas the 95% confidence area in which a new study will be located is represented by the dotted line.

The TP, FP, FN, TN, paired sensitivity and specificity, and the cutoff values of individual studies for urinary [TIMP-2] × [IGFBP7] levels to predict AKI are listed in Supplementary Table 1. In our analysis, the SROC curve did not show a typical ‘shoulder arm-shaped’ style, and the Spearman correlation coefficient was 0.098 (P = 0.682), indicating a lack of a threshold effect. The pooled sensitivity was 0.79 (95% CI 0.72, 0.84) and pooled specificity was 0.70 (95% CI 0.62, 0.76; Fig. 3). Pooled PLR was 2.6 (95% CI 2.1, 3.3), NLR was 0.31 (95% CI 0.23, 0.40), and pooled DOR was 8 (95% CI 6, 13). The AUROC was 0.81 (95% CI 0.78–0.84; Fig. 3). The I^2^ values for pooled sensitivity and specificity were 75.72 (95% CI 65.24, 86.20) and 93.29 (95% CI 91.29, 95.30), respectively. The Fagan nomogram (Supplementary Fig. 1) and likelihood ratio scatter plot (Supplementary Fig. 2) were used to estimate the diagnostic value and clinical availability of urinary [TIMP-2] × [IGFBP7] for AKI. The pooled pretest probability was 20%. The results showed that when [TIMP-2] × [IGFBP7] was positive, the post-test probability of [TIMP-2] × [IGFBP7] for AKI increased to 39%. In contrast, when the results were negative, the post-test probability of AKI detection dropped to 7%. The observed PLR value of 2.6 indicated that patients with AKI are 2.6 times more likely to have a positive diagnosis than healthy subjects, and an NLR of 0.31 suggested that the combination of TIMP-2 and IGFBP7 is a useful biomarker for the diagnosis of AKI.

### Subgroup analysis

To further investigate the value of [TIMP-2] × [IGFBP7] for the diagnosis of AKI among different age groups, diagnostic criteria, assessment times, clinical settings, and cut-off value. we performed a series of subgroup analyses. As one of the 20 studies enrolled infants, we reanalyzed the data of adults only in the age subgroup analysis. Sensitivity and specificity did not differ from the summary results in the adult group (0.79 and 0.71). The AUROC was 0.82. The 19 studies were divided into two subgroups according to age (65 years), and there was no significant difference in the predictive performance of urinary [TIMP-2] × [IGFBP7] among different age groups. Fifteen studies used all stages of kidney disease as the AKI diagnosis criteria, and five studies adopted the Stage 2–3 KDIGO criteria to define the AKI endpoint. The results suggested that urine [TIMP-2] × [IGFBP7] showed a better trend of sensitivity 0.84 (95% CI 0.73, 0.91), specificity 0.72 (95% CI 0.56, 0.84), and AUROC 0.86 (95% CI 0.83, 0.89) in the Stage 2–3 subgroup than in the all stage subgroup [sensitivity 0.77 (95% CI 0.69, 0.84), specificity 0.69 (95% CI 0.60, 0.77), and AUROC 0.80 (95% CI 0.76, 0.83)]. We divided the studies into three subgroups based on different measurement times (within 12 h, 12–24 h and 24–48 h). There were 9 studies in the “Within 12 h of measurement” subgroup; estimated AUROC was 0.78, while pooled sensitivity and specificity were 0.81 (95% CI 0.73 0.86) and 0.62 (95% CI 0.53, 0.70). There were 4 studies in the “12–24 h of measurement” subgroup; estimated AUROC was 0.80, while pooled sensitivity and specificity were 0.81 (95% CI 0.66, 0.90) and 0.67 (95% CI 0.54, 0.78). Additionally, our subgroup analysis found that seven studies with a measurement time of 24-48 h demonstrated a high level of diagnostic accuracy for AKI (sensitivity = 0.74, specificity = 0.82, AUC = 0.86) and an earlier diagnosis compared to current AKI diagnostic standards. Our study suggests that measuring urinary [TIMP-2] × [IGFBP7] within 24–48 h of admission may enable earlier identification of AKI, potentially improving patient outcomes. Nine studies focused on cardiac surgery patients, divided into “cardiac surgery” subgroups. Five others focused on patients in the ICU and two on patients in the ED and constituted the “ICU and ED” subgroup. Urine [TIMP-2] × [IGFBP7] showed good diagnostic value in patients undergoing cardiac surgery with an estimated AUROC of 0.83 (95% CI 0.79, 0.86). Pooled sensitivity and specificity were 0.75 (95% CI 0.64, 0.83) and 0.78 (95% CI 0.70, 0.84). Subgroup analysis was conducted based on the [TIMP-2] × [IGFBP7] cut-off value in urine, resulting in two groups: cut-off value equal to 0.3 and cut-off value not equal to 0.3. Our findings suggest that the latter group exhibited superior diagnostic performance (sensitivity = 0.78, specificity = 0.76, AUC = 0.82). The results of subgroup analyses are presented in Table 2.

**Table 2:** Results of subgroup analysis based on different standards.

Group		Sensitivity (95% CI)	Specificity (95% CI)	I^2^	PLR (95% CI)	NLR (95% CI)	DOR (95% CI)	AUROC (95% CI)
20 studies		0.79 (0.72–0.84)	0.70 (0.62–0.76)	97	2.6 (2.1–3.3)	0.31 (0.23–0.40)	8 (6–13)	0.81 (0.78–0.84)
Age	Mean age ≥ 65y (n = 9)	0.76 (0.65–0.83)	0.72 (0.61–0.81)	0	2.7 (1.7–4.1)	0.34 (0.22–0.54)	8 (3–18)	0.8 (0.77–0.83)
	Mean age < 65y (n = 10)	0.81 (0.74–0.87)	0.70 (0.58–0.79)	97	2.7 (2.0–3.6)	0.27 (0.20–0.36)	10 (7–15)	0.83 (0.80–0.86)
Criterion	All stage (n = 15)	0.77 (0.69–0.84)	0.69 (0.60–0.77)	97	2.5 (1.9–3.2)	0.33 (0.24–0.45)	8 (5–12)	0.8 (0.76–0.83)
	Stage 2–3 (n = 5)	0.84 (0.73–0.91)	0.72 (0.56–0.84)	40	3.0 (1.8–5.0)	0.23 (0.12–0.41)	13 (5–37)	0.86 (0.83–0.89)
Time of measurement	Within 12 h (n = 9)	0.81 (0.73–0.86)	0.62 (0.53–0.70)	93	2.1 (1.7–2.6)	0.32 (0.23–0.43)	7 (4–10)	0.78 (0.74–0.82)
	12-24 h (n = 4)	0.81 (0.66–0.90)	0.67 (0.54–0.78)	0	2.4 (1.5–3.9)	0.29 (0.14–0.60)	8 (3–27)	0.80 (0.76–0.83)
	24-48 h (n = 7)	0.74 (0.59–0.85)	0.82 (0.73–0.88)	38	4.1 (2.5–6.7)	0.31 (0.18–0.54)	13 (5–34)	0.86 (0.82–0.88)
Clinical setting	Cardiac surgery (n = 10)	0.73 (0.63–0.81)	0.76 (0.68–0.83)	68	3.1 (2.1–4.5)	0.35 (0.24–0.51)	9 (5–17)	0.81 (0.77–0.84)
	ICU and ED (n = 7)	0.83 (0.75–0.89)	0.54 (0.48–0.61)	88	1.8 (1.6–2.1)	0.32 (0.22–0.46)	6 (4–9)	0.73 (0.69–0.77)
	Others (n = 3)	0.80 (0.71–0.87)	0.79 (0.72–0.85)		4.0 (3.0–5.4)	0.24 (0.09–0.59)	25 (6–110)	0.9 (0.80–0.99)
Cut-off value	= 0.3 (n = 11)	0.79 (0.70–0.86)	0.62 (0.53–0.70)	96	2.1 (1.7–2.6)	0.34 (0.23–0.49)	6 (4–11)	0.76 (0.72–0.80)
	≠ 0.3 (n = 10)	0.78 (0.71–0.83)	0.76 (0.66–0.84)	81	3.3 (2.3–4.7)	0.29 (0.22–0.38)	11 (7–19)	0.82 (0.79–0.86)

*AUROC* area under the receiver operating characteristic curve, *CI* confidence interval, *DOR* diagnostic odds ratio, *ED* emergency department, *ICU* intensive care unit, *PLR* positive likelihood ratio, *NLR* negative likelihood ratio.

## Discussion

AKI is a common and severe clinical condition. Currently, the diagnosis of AKI is based on serum creatinine level and urine output; however, these diagnoses are insensitive, especially in the early stages of AKI. Serum creatinine levels do not rise until 24 to 72 h after kidney injury, and urine output is less specific and is also affected by diuretics. Over the past decades, many biomarkers, including neutrophil gelatinase-associated lipocalin (NGAL), kidney injury molecule 1 (KIM-1), and interleukin 18 (IL18), have been studied for the early diagnosis of AKI, but their diagnostic sensitivity is relatively low^36–38^. In recent years, urinary [TIMP-2] × [IGFBP7] has shown the best accuracy and stability in adult ICU patients who developed ischemic or nephrotoxic AKI. We conducted this meta-analysis, which included 20 studies (3625 patients) reporting the diagnostic value of urinary [TIMP-2] × [IGFBP7] in patients with different clinical settings of AKI. We found that this biomarker test had good diagnostic accuracy (sensitivity = 0.79, specificity = 0.70, AUC = 0.81) in all-cause AKI. In addition, the pooled DOR was 8 (95% CI 6–13), which suggests that urinary [TIMP-2] × [IGFBP7] had good diagnostic effectiveness for AKI. When the DOR is greater than 1, a higher diagnostic odds ratio indicates better diagnostic performance. For a DOR of less than 1, the reverse is true^39^. Moreover, it showed a better trend of sensitivity and specificity in patients with severe AKI (KDIGO stage 2–3) and in patients measured after 12 h. This result is similar to that of previous meta-analyses on this topic, which included 10 full-text prospective studies showing a sensitivity of 0.77, specificity of 0.76 and AUC of 0.83 for the early diagnosis of cardiac surgery-associated AKI in urine [TIMP-2] × [IGFBP7]^40^. There have been many studies in various clinical settings for AKI (e.g. surgery-associated, cardiac arrest-associated, sepsis) and age groups (e.g. older adults vs. younger adults) were included in the analysis.

Our study demonstrated that across various clinical contexts, a cutoff value of 0.3 was selected in 11 studies included in our analysis. When considering a predictive biomarker, it is crucial to establish a cutoff value that differentiates between disease and healthy groups. Unfortunately, we were unable to determine the optimal cutoff points for urine [TIMP-2] × [IGFBP7] due to the observed variation in cutoff values across the included studies. The large Sapphire and Opal cohorts have validated the cutoffs of 0.3 and 2.0 for moderate-to-severe AKI^18,19^. However, it remains an open question as to how clinicians can effectively utilize this potential in different clinical contexts.

TIMP-2 and IGFBP7 are two biomarkers of G1 phase cell cycle arrest. These factors act by blocking the binding of cyclin and protein kinases through regulation of the P53 and P21 signaling pathways and alteration of the response of cells to inflammatory factors and toxins^41^. AKI-induced increases in urinary TIMP2 and IFGBP7 are caused by increased filtration, reduced tubule reabsorption, and urinary leakage of TIMP2 and IGFBP7 from proximal tubule cells^42^. Such physiological and pathological mechanisms lead to increased urinary TIMP2 and IGFBP7 levels in acute kidney injury and their role in the early prediction of AKI. Our studies suggested that urinary [TIMP-2] × [IGFBP7] showed excellent diagnostic value in patients undergoing cardiac surgery, with an estimated AUC of 0.81. The pooled sensitivity and specificity were 0.73 and 0.76. Zaouter et al. found that in a population at risk of developing cardiac surgery-associated AKI (CSA-AKI), urinary [TIMP-2] × [IGFBP7] was not detected in the first postoperative week within the first 24 postoperative hours^29^. Conversely, in Levante’s study, [TIMP-2] × [IGFBP7] had an AUC of 0.92 for predicting AKI after cardiac surgery, and a sensitivity and specificity of 0.84 and 0.88, respectively, for a cutoff value of 0.3^43^. Several studies have investigated the ability of urinary [TIMP-2] × [IGFBP7] to predict AKI after cardiac surgery. However, due to the different AKI definitions, measurement times, and cutoff values, the results are not consistent; therefore, further large-scale studies are required.

Significant heterogeneity was observed in this meta-analysis. In subgroup analysis, inter-study heterogeneity may be derived from the AKI threshold. The AUC was higher in the KDIGO stage 2–3 subgroup than in all KDIGO stage subgroups (0.86 vs. 0.80). Similarly, Su et al. showed that urine [TIMP-2] × [IGFBP7] had the best test characteristics for KDIGO stage 2–3^40^. However, Jia et al. suggested that the AUC was higher in the KDIGO Stage 1 subgroup^44^, but there were only three studies in the “KDIGO stage 1” subgroup. AKI diagnosed by the KDIGO stage 1 criteria is affected by a variety of factors, including blood concentration and drugs; thus, it may only reflect pure functional impairment, without a true kidney injury having occured. TIMP-2 and IGFBP7 levels in the urine were elevated only when the kidney was in emergency or damaged conditions. Therefore, urine [TIMP-2] × [IGFBP7] may be more sensitive for predicting severe AKI (KDIGO stage 2 or 3).

Despite significant advances in the epidemiology of AKI, predicting kidney recovery after AKI remains a major clinical challenge. One study has shown that plasma NGAL can predict AKI recovery, but its predictive performance is limited^45^. In another study, it was shown that the course of urinary [TIMP-2] × [IGFBP7] concentration predicted renal recovery from AKI following cardiac surgery (AUC: 0.79, 95% CI 0.65, 0.92). In contrast to [TIMP-2] × [IGFBP7], urinary NGAL concentration failed to predict renal recovery (AUC: 0.48, 95% CI 0.31, 0.64)^20^. The correlation between cell cycle arrest biomarkers and AKI outcomes deserves further investigation. Our data are in line with Sakyi’s study that urinary [TIMP-2] × [IGFBP7] showed the best diagnostic performance in predicting KDIGO AKI stage 2 and 3. Moreover, Sakyi et al. also suggested mean level of [TIMP-2] × [IGFBP7] increased as AKI stage increased^32^. The levels of cell cycle arrest biomarkers ([TIMP-2] × [IGFBP7]) increased with increasing AKI severity, indicating it could be used to monitor the progress of the condition. However, the severity of AKI is associated with a significantly increased incidence of clinically important outcomes such as renal replacement therapy, in-hospital mortality, and persistent renal dysfunction. Consequently, early detection and risk assessment could improve patient outcomes through early intervention and optimized patient management.

The present study had several limitations. First, some of the included studies had small sample sizes, which may have led to an overestimation of urinary [TIMP-2] × [IGFBP7] in the diagnosis of AKI. Second, most of the published literature on [TIMP-2] × [IGFBP7] has focused on its application in adult ICU patients and cardiac surgery patients. Whether [TIMP-2] × [IGFBP7] use can be expanded to other contexts or patient populations, such as pediatric patients, has not been confirmed. Finally, many of the included studies assessed urine [TIMP-2] × [IGFBP7] at a single point in time, without continuous measurements, and lacked predictions of AKI progression and prognosis.

## Conclusion

In conclusion, this meta-analysis included updated clinical studies and used more accurate analysis methods to assess the diagnostic value of urine [TIMP-2] × [IGFBP7] compared to the current literature. Our meta-analysis suggests that urinary [TIMP-2] × [IGFBP7] levels have good predictive value as biomarkers of AKI in a wide range of clinical settings. However, whether and how urinary [TIMP-2] × [IGFBP7] can be widely used in the clinical diagnosis of all-cause AKI needs to be studied in different clinical settings, patient populations, and disease spectrum studies in the future.

## Supplementary Information

Supplementary Information 1.

Supplementary Information 2.

## Data Availability

All data relevant to the study are included in the article or uploaded as Supplementary Information. In addition, the datasets used and/or analyzed during the current study are available from the corresponding author on reasonable request.

## References

[1] Susantitaphong P, Cruz DN, Cerda J, Abulfaraj M, Alqahtani F, Koulouridis I (2013). World incidence of AKI: A meta-analysis. Clin. J. Am. Soc. Nephrol..

[2] Yang L, Xing G, Wang L, Wu Y, Li S, Xu G (2015). Acute kidney injury in China: A cross-sectional survey. Lancet.

[3] Tang X, Chen D, Yu S, Yang L, Mei C (2017). Consortium IAbC: Acute kidney injury burden in different clinical units: Data from nationwide survey in China. PLoS ONE.

[4] Roy AK, Mc Gorrian C, Treacy C, Kavanaugh E, Brennan A, Mahon NG (2013). A comparison of traditional and novel definitions (RIFLE, AKIN, and KDIGO) of acute kidney injury for the prediction of outcomes in acute decompensated heart failure. Cardiorenal Med..

[5] Thomas ME, Blaine C, Dawnay A, Devonald MA, Ftouh S, Laing C (2015). The definition of acute kidney injury and its use in practice. Kidney Int..

[6] Lane BR, Poggio ED, Herts BR, Novick AC, Campbell SC (2009). Renal function assessment in the era of chronic kidney disease: Renewed emphasis on renal function centered patient care. J. Urol..

[7] Lane BR, Demirjian S, Weight CJ, Larson BT, Poggio ED, Campbell SC (2010). Performance of the chronic kidney disease-epidemiology study equations for estimating glomerular filtration rate before and after nephrectomy. J. Urol..

[8] Sreeram GM, Grocott HP, White WD, Newman MF, Stafford-Smith M (2004). Transcranial Doppler emboli count predicts rise in creatinine after coronary artery bypass graft surgery. J. Cardiothorac. Vasc. Anesth..

[9] Emlet DR, Pastor-Soler N, Marciszyn A, Wen X, Gomez H, Humphries WH (2017). Insulin-like growth factor binding protein 7 and tissue inhibitor of metalloproteinases-2: Differential expression and secretion in human kidney tubule cells. Am. J. Physiol. Renal Physiol..

[10] Kashani K, Al-Khafaji A, Ardiles T, Artigas A, Bagshaw SM, Bell M (2013). Discovery and validation of cell cycle arrest biomarkers in human acute kidney injury. Crit. Care.

[11] Oezkur M, Magyar A, Thomas P, Stork T, Schneider R, Bening C (2017). TIMP-2*IGFBP7 (Nephrocheck (R)) measurements at intensive care unit admission after cardiac surgery are predictive for acute kidney injury within 48 hours. Kidney Blood Press. Res..

[12] Whiting P, Rutjes AW, Reitsma JB, Bossuyt PM, Kleijnen J (2003). The development of QUADAS: A tool for the quality assessment of studies of diagnostic accuracy included in systematic reviews. BMC Med. Res. Methodol..

[13] Higgins JP, Thompson SG, Deeks JJ, Altman DG (2003). Measuring inconsistency in meta-analyses. BMJ.

[14] Liang Z, Zhang Q, Wang C, Shi F, Cao H, Yu Y (2017). Hyaluronic acid/ Hyaluronidase as biomarkers for bladder cancer: A diagnostic meta-analysis. Neoplasma.

[15] Deeks JJ, Macaskill P, Irwig L (2005). The performance of tests of publication bias and other sample size effects in systematic reviews of diagnostic test accuracy was assessed. J. Clin. Epidemiol..

[16] Schiefer J, Lichtenegger P, Berlakovich GA, Plochl W, Krenn CG, Baron DM (2019). Urinary [TIMP-2] x [IGFBP-7] for predicting acute kidney injury in patients undergoing orthotopic liver transplantation. BMC Nephrol..

[17] Fuhrman DY, Kellum JA, Joyce EL, Miyashita Y, Mazariegos GV, Ganoza A (2020). The use of urinary biomarkers to predict acute kidney injury in children after liver transplant. Pediatr. Transplant..

[18] Bihorac A, Chawla LS, Shaw AD, Al-Khafaji A, Davison DL, Demuth GE (2014). Validation of cell-cycle arrest biomarkers for acute kidney injury using clinical adjudication. Am. J. Respir. Crit. Care Med..

[19] Hoste EA, McCullough PA, Kashani K, Chawla LS, Joannidis M, Shaw AD (2014). Derivation and validation of cutoffs for clinical use of cell cycle arrest biomarkers. Nephrol. Dial. Transplant..

[20] Meersch M, Schmidt C, Van Aken H, Martens S, Rossaint J, Singbartl K (2014). Urinary TIMP-2 and IGFBP7 as early biomarkers of acute kidney injury and renal recovery following cardiac surgery. PLoS ONE.

[21] Pilarczyk K, Edayadiyil-Dudasova M, Wendt D, Demircioglu E, Benedik J, Dohle DS (2015). Urinary [TIMP-2]*[IGFBP7] for early prediction of acute kidney injury after coronary artery bypass surgery. Ann. Intens. Care.

[22] Dusse F, Edayadiyil-Dudasova M, Thielmann M, Wendt D, Kahlert P, Demircioglu E (2016). Early prediction of acute kidney injury after transapical and transaortic aortic valve implantation with urinary G1 cell cycle arrest biomarkers. BMC Anesthesiol..

[23] Honore PM, Nguyen HB, Gong M, Chawla LS, Bagshaw SM, Artigas A (2016). Urinary tissue inhibitor of metalloproteinase-2 and insulin-like growth factor-binding protein 7 for risk stratification of acute kidney injury in patients with sepsis. Crit. Care Med..

[24] Kimmel M, Shi J, Latus J, Wasser C, Kitterer D, Braun N (2016). Association of renal stress/damage and filtration biomarkers with subsequent AKI during hospitalization among patients presenting to the emergency department. Clin. J. Am. Soc. Nephrol..

[25] Finge T, Bertran S, Roger C, Candela D, Pereira B, Scott C (2017). Interest of urinary [TIMP-2] x [IGFBP-7] for predicting the occurrence of acute kidney injury after cardiac surgery: A gray zone approach. Anesth. Analg..

[26] Mayer T, Bolliger D, Scholz M, Reuthebuch O, Gregor M, Meier P (2017). Urine biomarkers of tubular renal cell damage for the prediction of acute kidney injury after cardiac surgery—A pilot study. J. Cardiothorac. Vasc. Anesth..

[27] Wang Y, Zou Z, Jin J, Teng J, Xu J, Shen B (2017). Urinary TIMP-2 and IGFBP7 for the prediction of acute kidney injury following cardiac surgery. BMC Nephrol..

[28] Adler C, Heller T, Schregel F, Hagmann H, Hellmich M, Adler J (2018). TIMP-2/IGFBP7 predicts acute kidney injury in out-of-hospital cardiac arrest survivors. Crit. Care.

[29] Zaouter C, Potvin J, Bats ML, Beauvieux MC, Remy A, Ouattara A (2018). A combined approach for the early recognition of acute kidney injury after adult cardiac surgery. Anaesth. Crit. Care Pain Med..

[30] Chen J, Sun Y, Wang S, Dai X, Huang H, Bai Z (2020). The effectiveness of urinary TIMP-2 and IGFBP-7 in predicting acute kidney injury in critically ill neonates. Pediatr. Res..

[31] Hatton GE, Wang YW, Isbell KD, Finkel KW, Kao LS, Wade CE (2020). Urinary cell cycle arrest proteins urinary tissue inhibitor of metalloprotease 2 and insulin-like growth factor binding protein 7 predict acute kidney injury after severe trauma: A prospective observational study. J. Trauma Acute Care Surg..

[32] Sakyi SA, Ephraim RKD, Adoba P, Amoani B, Buckman T, Mantey R (2021). Tissue inhibitor metalloproteinase 2 (TIMP-2) and insulin-like growth factor binding protein 7 (IGFBP7) best predicts the development of acute kidney injury. Heliyon.

[33] Waskowski J, Pfortmueller CA, Schenk N, Buehlmann R, Schmidli J, Erdoes G (2021). (TIMP2) x (IGFBP7) as early renal biomarker for the prediction of acute kidney injury in aortic surgery (TIGER). A single center observational study. PLoS ONE.

[34] Irqsusi M, Beckers J, Wiesmann T, Talipov I, Ramzan R, Rastan AJ (2022). Urinary TIMP-2 and IGFBP-7 protein levels as early predictors of acute kidney injury after cardiac surgery. J. Card. Surg..

[35] Yang HS, Hur M, Lee KR, Kim H, Kim HY, Kim JW (2022). Biomarker rule-in or rule-out in patients with acute diseases for validation of acute kidney injury in the emergency department (BRAVA): A multicenter study evaluating urinary TIMP-2/IGFBP7. Ann. Lab. Med..

[36] Haase M, Bellomo R, Devarajan P, Schlattmann P, Haase-Fielitz A (2009). Group NM-aI: Accuracy of neutrophil gelatinase-associated lipocalin (NGAL) in diagnosis and prognosis in acute kidney injury: A systematic review and meta-analysis. Am. J. Kidney Dis..

[37] Huang Y, Don-Wauchope AC (2011). The clinical utility of kidney injury molecule 1 in the prediction, diagnosis and prognosis of acute kidney injury: A systematic review. Inflamm. Allergy Drug Targets.

[38] Liu Y, Guo W, Zhang J, Xu C, Yu S, Mao Z (2013). Urinary interleukin 18 for detection of acute kidney injury: A meta-analysis. Am. J. Kidney Dis..

[39] Glas AS, Lijmer JG, Prins MH, Bonsel GJ, Bossuyt PM (2003). The diagnostic odds ratio: A single indicator of test performance. J. Clin. Epidemiol..

[40] Su LJ, Li YM, Kellum JA, Peng ZY (2018). Predictive value of cell cycle arrest biomarkers for cardiac surgery-associated acute kidney injury: A meta-analysis. Br. J. Anaesth..

[41] Zuo S, Liu C, Wang J, Wang F, Xu W, Cui S (2012). IGFBP-rP1 induces p21 expression through a p53-independent pathway, leading to cellular senescence of MCF-7 breast cancer cells. J. Cancer Res. Clin. Oncol..

[42] Johnson ACM, Zager RA (2018). Mechanisms underlying increased TIMP2 and IGFBP7 urinary excretion in experimental AKI. J. Am. Soc. Nephrol..

[43] Levante C, Ferrari F, Manenti C, Husain-Syed F, Scarpa M, Hinna Danesi T (2017). Routine adoption of TIMP2 and IGFBP7 biomarkers in cardiac surgery for early identification of acute kidney injury. Int. J. Artif. Organs.

[44] Jia HM, Huang LF, Zheng Y, Li WX (2017). Diagnostic value of urinary tissue inhibitor of metalloproteinase-2 and insulin-like growth factor binding protein 7 for acute kidney injury: A meta-analysis. Crit. Care.

[45] Srisawat N, Murugan R, Lee M, Kong L, Carter M, Angus DC (2011). Plasma neutrophil gelatinase-associated lipocalin predicts recovery from acute kidney injury following community-acquired pneumonia. Kidney Int..

