# Supplementary material for: Predictive value of urinary cell cycle arrest biomarkers for all cause-acute kidney injury: a meta-analysis
Source: Sci Rep. 2023 Apr 13;13:6037. doi: 10.1038/s41598-023-33233-9 (PMC10102152; doi:10.1038/s41598-023-33233-9)
Supplement: Supplementary file 2 — Supplementary Information 2. [file 41598_2023_33233_MOESM2_ESM.docx]

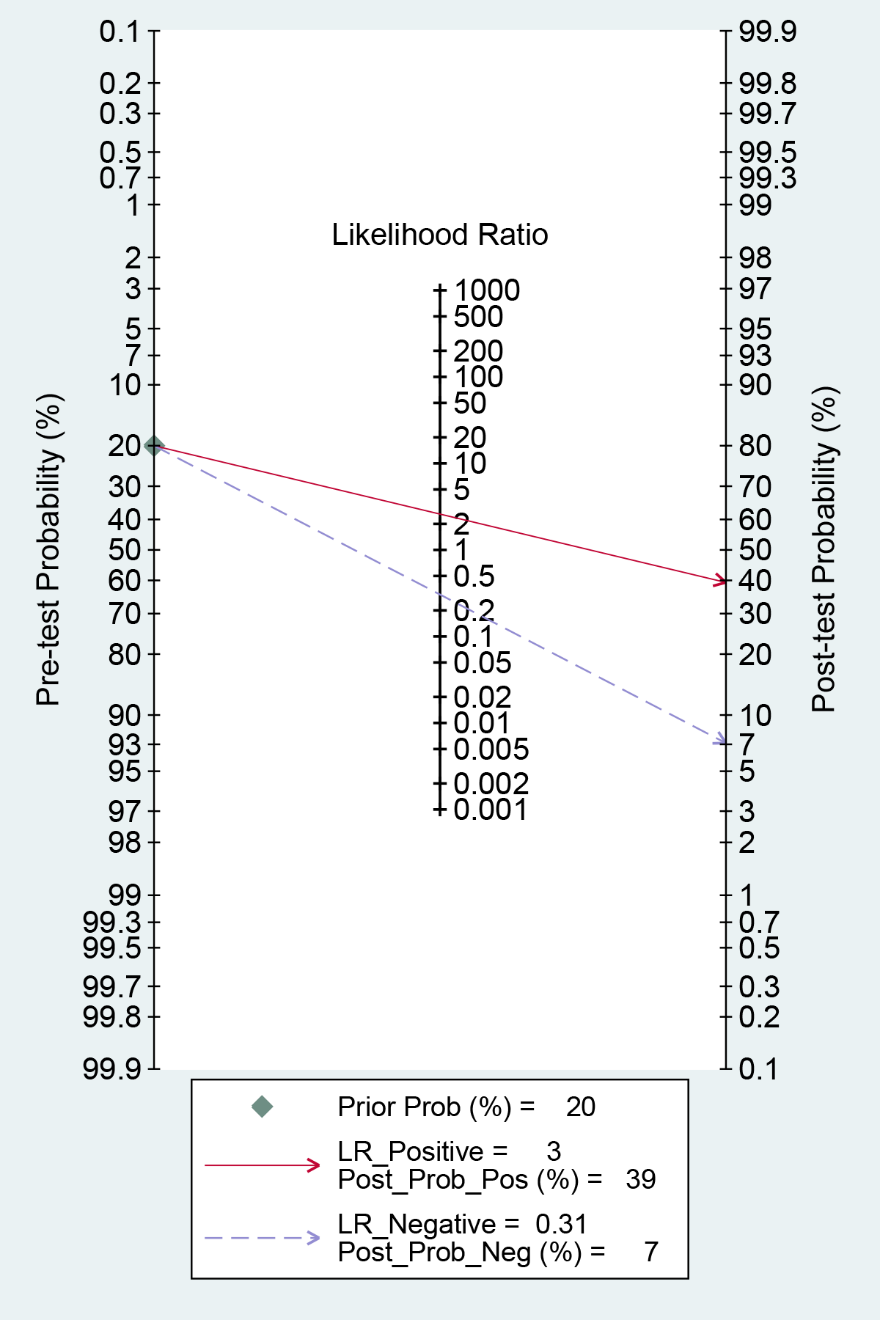


**Supplementary Fig 1** Fagan nomogram of urinary [TIMP-2]·[IGFBP7] for the diagnosis of

acute kidney injury.


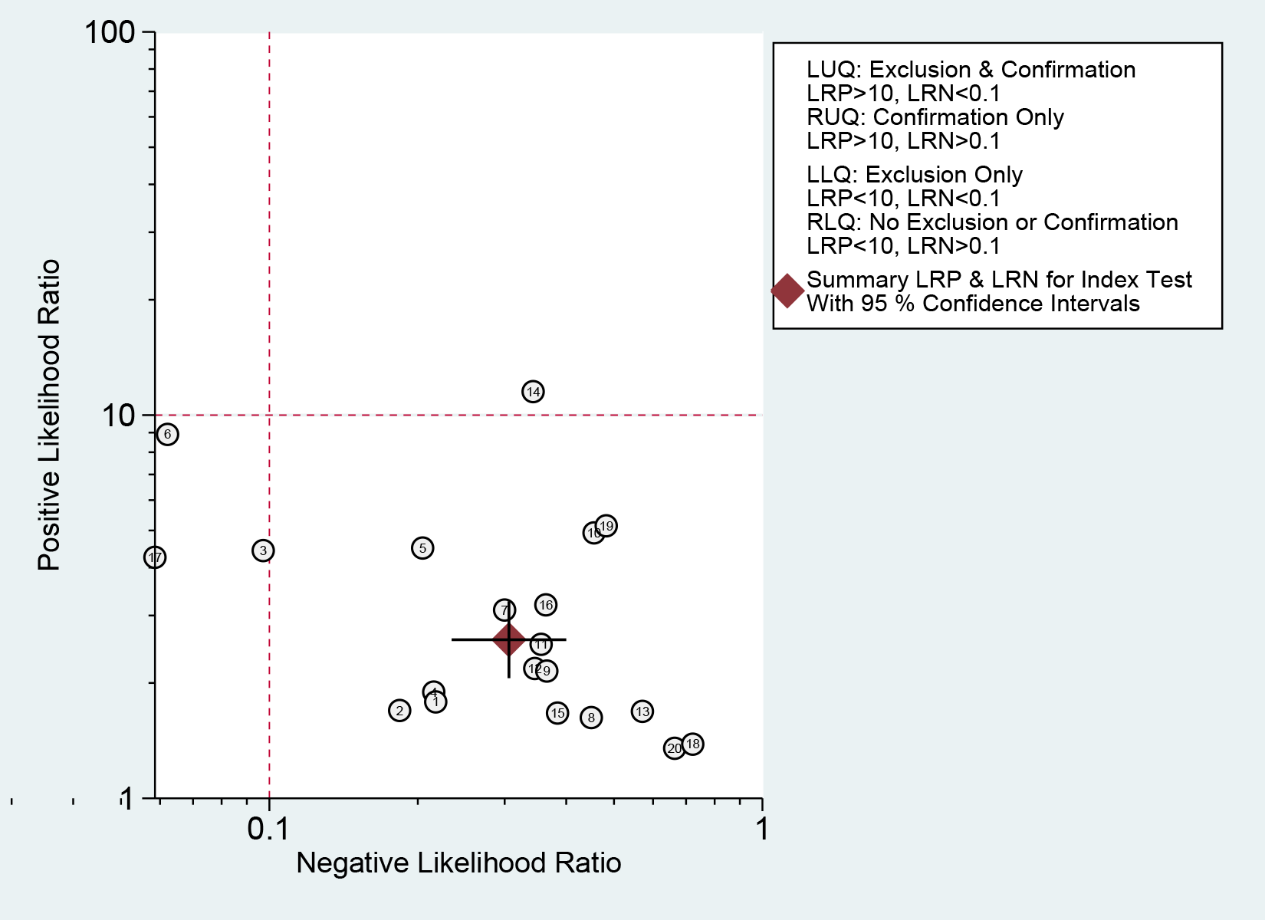


**Supplementary Fig 2** Likelihood ratio scattergram of urinary [TIMP-2]·[IGFBP7] for the diagnosis of acute kidney injury. The positive likelihood ratio and negative likelihood ratio were 2.6 (95% CI 2.1–3.3) and 0.31 (95% CI 0.23–0.40), respectively. CI=confidence interval, LLQ=left lower quadrant, LRN=likelihood ratio negative, LRP=likelihood ratio positive, LUQ=left upper quadrant, RLQ= right lower quadrant, RUQ=right upper quadrant.

| **Supplementary Table 1** Diagnostic value of urinary [TIMP-2] × [IGFBP7] for acute kidney injury in individual studies | **Meauring time** | Within 12h of ICU admission | Within 12h of ICU admission | 24h after surgery | on ICU admission | 1 day after surgery | 24h after TAVI | Within 12 hours | Within 27 hours after enrollment | 3h post CPB | 24 h post CPB | 4h post cardiac surgery | 1h post CPB |
| --- | --- | --- | --- | --- | --- | --- | --- | --- | --- | --- | --- | --- | --- |
|  | **Cut-off value** | 0.3 | 0.3 | 0.5 | 0.3 | 0.82 | 1.03 | 1 | 0.3 | 0.3 | 0.3 | 0.3 | 0.41 |
|  | **AUROC(95% CI)** | 0.8(NR) | 0.82(0.76-0.88) | 0.84(NR) | 0.79(0.69–0.88) | 0.82(0.622–1.0) | 0.97 (0.914-1.0) | 0.84(0.77-0.90) | 0.76(0.68- 0.84) | 0.73(0.62-0.83) | 0.75(NR) | 0.80(0.68-0.91) | 0.78(0.67-0.839) |
|  | **Specificity** | 50% | 46% | 81% | 53% | 81% | 90% | 75% | 53% | 64% | 88% | 70% | 64% |
|  | **Sensitivity** | 89% | 92% | 92% | 89% | 89% | 100% | 76% | 76% | 76% | 60% | 75% | 78% |
|  | **TN** | 314 | 155 | 19 | 49 | 44 | 29 | 144 | 134 | 38 | 101 | 26 | 65 |
|  | **FN** | 11 | 6 | 2 | 7 | 1 | 0 | 9 | 11 | 8 | 14 | 5 | 2 |
|  | **FP** | 313 | 182 | 5 | 43 | 10 | 3 | 48 | 118 | 21 | 14 | 11 | 36 |
|  | **TP** | 90 | 65 | 24 | 54 | 5 | 8 | 31 | 35 | 26 | 21 | 15 | 7 |
|  | **Stydy** | Kashani(2013)**^【10】^** | Bihorac(2014) **^【18】^** | Meersch(2014)**^【20】^** | Hoste(2014)**^【19】^** | Pilarczy(2015)**^【21】^** | Dusse(2016) **^【22】^** | Honore(2016) **^【23】^** | Kimme(2016)**^【24】^** | Finge(2017)**^【25】^** | Oezkur(2017)**^【11】^** | Wang(2017) **^【27】^** | Mayer(2017) **^【26】^** |

| **Supplementary Table 1** (continued) | **Meauring time** | 12h after surgery | 3 h after determination of OHCA | at the end of OLT | 24h after OLT | 48h after OLT | 6h after liver transplanet | 2 weeks after NICU asmission | Within 48h of trauma ICU admission | Within 48h of in-patient | 24h after abdominal aortic surgery | 0 h after admission to ICU | 24h after admission to ICU | Abbreviations: AUROC, area under the receiver operating characteristic curve; CPB, cardiopulmonary bypass; FP, false positive; FN, false negative; ICU, intensive care unit; NICU, neonatal intensive care unit; NR, not reported; OHCA, out-of-hospital cardiac arrest; OLT, orthotopic liver transplantation; TAVI, transcatheter aortic valve implantation; TN, true negative; TP, true positive; Cut-off value, ng/mL^2^/1000. |
| --- | --- | --- | --- | --- | --- | --- | --- | --- | --- | --- | --- | --- | --- | --- |
|  | **Cut-off value** | 0.3 | 0.24 | NR | NR | NR | NR | 0.045 | 0.33 | 0.3 | 0.3 | 0.07 | 0.35 |  |
|  | **AUROC(95% CI)** | 0.69(0.53-0.84) | 0.97(0.90-1.00) | 0.54(0.32-0.75) | 0.60(0.41-0.79) | 0.63(0.46-0.8) | 0.93(0.81-1.00) | 0.65(0.49-0.75)) | 0.73(0.65-0.84) | 0.94(0.90-0.98) | 0.6(0.49-0.71) | 0.725(NR) | 0.718(NR) |  |
|  | **Specificity** | 0.62 | 0.941 | NR | NR | NR | NR | 52.10% | 78.00% | 77.20% | 58.00% | 55.60% | 88.20% |  |
|  | **Sensitivity** | 0.65 | 0.968 | NR | NR | NR | NR | 80.00% | 72.00% | 95.80% | 58.00% | 84.60% | 53.80% |  |
|  | **TN** | 8 | 16 | NR | NR | NR | NR | 113 | 38 | 83 | 36 | 20 | 32 |  |
|  | **FN** | 13 | 10 | NR | NR | NR | NR | 4 | 11 | 2 | 13 | 2 | 6 |  |
|  | **FP** | 5 | 1 | NR | NR | NR | NR | 104 | 11 | 24 | 26 | 16 | 4 |  |
|  | **TP** | 24 | 21 | NR | NR | NR | NR | 16 | 28 | 42 | 18 | 12 | 8 |  |
|  | **Stydy** | Zaouter(2018) **^【29】^** | Adler(2018)**^【28】^** | Schiefer(2019)**^【16】^** |  |  | Fuhrman(2020)**^【17】^** | Chen(2020)**^【30】^** | Hatton(2020)**^【31】^** | Sakyi(2021)**^【32】^** | Waskowski(2021)**^【33】^** | Irqsusi(2021)**^【34】^** |  |  |
